# Validation of the Breastfeeding Score—A Simple Screening Tool to Predict Breastfeeding Duration

**DOI:** 10.3390/nu11122852

**Published:** 2019-11-21

**Authors:** Hanne Kronborg, Michael Væth

**Affiliations:** Department of Public Health, Faculty of Health, Aarhus University, 8000 Aarhus, Denmark; vaeth@ph.au.dk

**Keywords:** breastfeeding, infant, parity, maternal behavior, educational status, self-efficacy, risk factors, prognosis, sensitivity and specificity, practice

## Abstract

Easy to use screening tools to identify mothers in risk of early breastfeeding cessation are needed. The purpose was to validate a revised version of the breastfeeding score, consisting of four questions addressing completed education, earlier breastfeeding duration, self-efficacy, and sense of security not knowing the exact amount of milk the baby ingests. We used two cohorts from 2004 (*n* = 633) and 2017 (*n* = 579) to explore the predictive validity of the breastfeeding score to identify mothers at risk of breastfeeding cessation within the first 17 weeks postpartum. The analyses included sensitivity and specificity, clinically relevant cut-points, and calibrations plots. A cut-point ≥5 points identified 61% of first-time and 42% of multiparous mothers in the validation cohort 2017 to be at risk of early breastfeeding cessation with a sensitivity and specificity of 80% and 60% for first-time, and 69% and 82% for multiparous, respectively. The corresponding numbers in the 2004 cohort were almost identical. The area under the receiver operating characteristic (ROC) curves were 0.77 and 0.78 and the calibration plots showed good agreement for the two cohorts. The breastfeeding score indicated good ability to discriminate between mothers at risk of early exclusive breastfeeding cessation. The simple form of the tool makes it easy to use in daily practice.

## 1. Introduction

Breastfeeding has been shown to be beneficial for the health of both the mother and infant, not only in the postnatal period, but also later in life [1]. However, breastfeeding rates are still below the recommended in western societies [2]. One third of new mothers experience that establishment of breastfeeding is complicated by early breastfeeding problems [3,4]. From ongoing Cochrane reviews, we know that professional support in the early postpartum period helps mothers to successfully establish breastfeeding [5]. The challenge is to identify the mothers at risk of early breastfeeding cessation and thus in need of early support. Usable screening tools have to be simple to use in practice and reliable to discriminate between mothers.

Successful breastfeeding depends on multiple factors [6,7,8], and a tool to predict breastfeeding duration should therefore include different kinds of indicators. The challenge is still to establish a link from the risk profile of predictors for early cessation of breastfeeding to a practical tool (index) providing valid information for professionals to initiate preventive efforts. Consensus has not yet been reached on which indicators or indexes are the most reliable. Numerous postpartum screening tools developed during the last decades include behavioral and observational factors connected to breastfeeding [9]; others include more self-reported psychosocial factors [10]. So far, the tools have primarily been evaluated for construct and content validity and psychometric properties, and, to a lesser extent, for their predictive validity [10,11,12,13]. Moreover, a number of tools are comprehensive and contain a large number of variables, making them less useful in practice [10,11,12,13]. The Breastfeeding Self-Efficacy Scale-Short Form (BSES-SF), including 14 items measuring perceived self-efficacy towards breastfeeding, has been the tool that has shown the best ability to predict breastfeeding success [12,14]. Still, the question remains whether it is possible to produce a reliable self-reported tool with fewer questions to predict early breastfeeding cessation.

In 2007, we introduced a breastfeeding score, developed on the basis of data from a cohort of breastfeeding mothers from 1999 and validated using cohort data from 2004 [15]. This breastfeeding score was based on four simple questions and showed a strong prediction of the breastfeeding cessation in the first four months postpartum. Thus, the tool complied with demands of both simplicity and reliability. The aim of the present study was to validate a revised version of the breastfeeding score using new and expanded data.

## 2. Materials and Methods

The validation process followed the method suggested by Altman et al. [16,17,18]. The breastfeeding score was first tested in a temporal validation on a second dataset collected independently of the first dataset which was used in the development process. In the present study, we also validated the revised version of the breastfeeding score on a third dataset of mothers from different geographical areas where we had no exclusion criteria.

### 2.1. Three Cohorts Included in the Derivation and Validation Process

The breastfeeding score was obtained from the derivation cohort collected in 1999 [19], which included 471 Danish-speaking mothers who had given birth to a single child with a gestational age ≥37 weeks. The temporal validation was performed on a cohort of 723 mothers from 2004 [20], which constituted the comparison group from a randomized trial with the same inclusion criteria and data obtained from the same area as the derivation cohort. Detailed description of the cohorts can be found elsewhere [19,20].

The external validation cohort from 2017 (*n* = 612) consisted of the comparison group in a community-based randomized trial [21,22]. The aim of the trial was to test the effect of the Newborn Behavioral Observation method with the comparison group treated as usual. The health visitor recruited mothers from the beginning of January 2017 until the end of January 2018 at the first home visit one to two weeks after birth. There were no exclusion criteria, except exclusion of mothers who were unable to manage own legal affairs. The validation cohort from 2017 thus included first-time mothers, premature births, twin births, postpartum depressed mothers, and mothers with a different ethnic background than Danish. The study was performed in four Danish municipalities, representing several geographical regions of Denmark [22]. The comparison groups were chosen as validation cohorts to avoid impact from the interventions studied in the randomized trials.

### 2.2. Data Collection

Data in all three cohorts were collected from self-reported questionnaires. Questions included in the breastfeeding score were identical in wording and were collected approximately two weeks postpartum. The duration of exclusive breastfeeding was defined as only ingesting mother’s milk according to indicators for assessing breastfeeding practices from the WHO [23]. Data on breastfeeding duration were collected by the local health visitors in the derivation cohort and self-reported in the validation cohorts. The follow-up periods were 17 and 26 weeks for the 1999 and 2004 cohorts, and nine months for the 2017 cohort. Data were obtained by mail in the first two cohorts, and by a web-based system in the 2017 cohort.

### 2.3. Ethical Approval

Permission to conduct the cohort studies in 1999 and 2004 was obtained from the Science Ethic Committee for the Counties of Ringkoebing, Ribe and Soenderjylland (ref. 2047-99) and (ref. 2480-03), and the Danish Data Protection Agency (j.nr. 2013-41-1866, 1344) and (j.nr. 2003-41-3306), respectively. According to the cohort study 2017 the Central Denmark Region Committee on Health Research Ethics found no need for ethical approval (ref.no.172/2016) as there was no biomedicine involved in the project. Written consent was obtained from all participating mothers. Approval was obtained from the Danish Data Protection Agency (ref.no. 62908/2016).

### 2.4. Risk Factors Included in the Breastfeeding Score

Originally, the breastfeeding score included the following four risk factors: duration of schooling, previous breastfeeding experience, self-efficacy concerning breastfeeding, and mother’s confidence not knowing the exact amount of milk the baby ingests when breastfeeding [15,19]. These factors represent information within sociodemographic, pre and perinatal as well as psychosocial domains.

In the current validation, we chose to change the sociodemographic variable from years of schooling to level of education. Originally, we used the distinction between having completed primary or secondary school (high school) [19]. Today, more than 70% finish secondary school in Denmark, and the level of further education is thus more informative. Furthermore, we changed the variable name from confidence to sense of security (not knowing the exact amount of milk the baby ingests when breastfeeding). The variable name was changed primarily to achieve a more descriptive translation of the content of the question and secondly to make a clearer distinction from the self-efficacy variable. The wording of the question (in Danish) was not changed. Thus, the current validation included the following four risk factors: Completed education measured at levels: none, short, skilled, theoretical bachelor, master. Earlier breastfeeding duration measured in weeks: none for primipara, 0–5, 6–17, >17 for multipara. Self-efficacy measured on a five-point Likert scale from very certain to very uncertain. Sense of security not knowing the exact amount of milk the baby ingests measured on a five-point Likert scale from very secure to very unsecure. Questions and their categorization appear from Table 1.

### 2.5. Statistical Methods

Initially, descriptive analyses of the risk factors in the three cohorts were performed, and Chi^2^ tests were used to compare the derivation and validation cohorts, as seen in Table 1. Next, we used Cox regression analysis to assess the influence of the four risk factors on exclusive breastfeeding cessation in the first 17 weeks postpartum in the derivation cohort. For comparison, data from the two validation cohorts were analysed in the same way. The hazard ratios (HR) estimated the ratio of the cessation rate of exclusive breastfeeding for a given category of the risk factor relative to the cessation rate for the reference category. Integer scores roughly proportional to the log (HR) estimates in the derivation cohort were subsequently obtained for each category of the four risk factors. Finally, for each mother, the breastfeeding score was computed as the sum of the scores for the categories to which she belonged, providing a possible risk score of 0–12 points, as seen in Table 2. The scoring thus obtained was subsequently validated on the two independently collected dataset to evaluate the robustness of the prediction of the breastfeeding score as suggested by Altman et al. [16,17].

**Figure 1:**
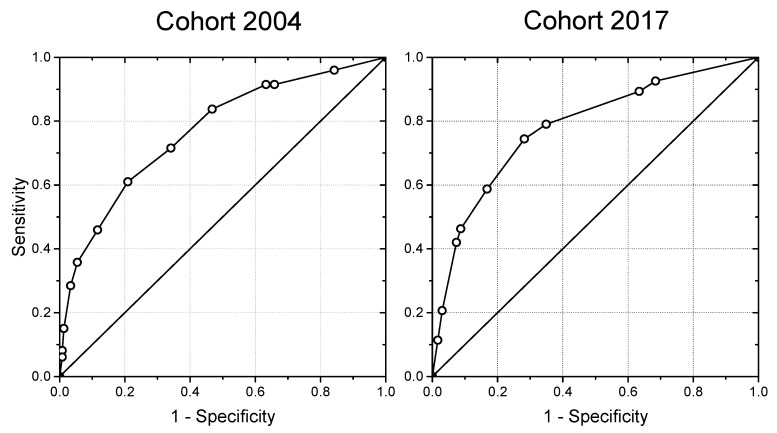
Receiver operating characteristic (ROC) curves for the breastfeeding scores in the validation 2004 (*n* = 633) and 2017 (*n* = 579) cohorts.

**Figure 2:**
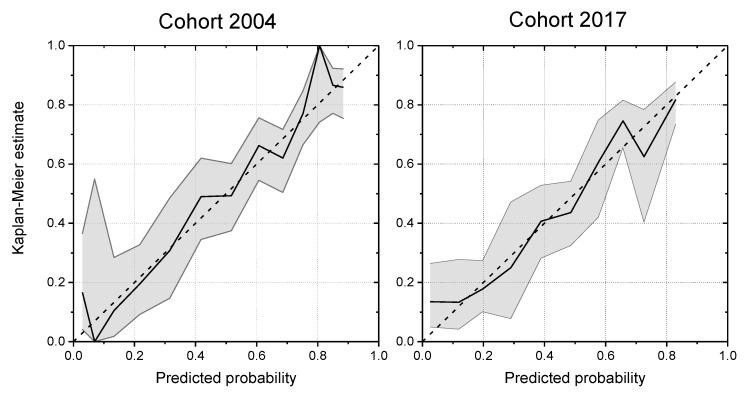
Calibrations plots with 95% confidence intervals for the breastfeeding scores in the validation cohorts 2004 (*n* = 633) and 2017 (*n* = 579).

The two validation cohorts were used to investigate the breastfeeding score’s ability to predict maternal risk of early breastfeeding cessation defined as cessation of exclusive breastfeeding before the end of week 17. Subsequently, we used receiver operating characteristic (ROC) curves to explore the diagnostic validity of the breastfeeding score and to identify a suitable cut-point, as seen in Figure 1. Moreover, the validity of the breastfeeding score was evaluated by calibration plots, as seen in Figure 2, and analyses, describing numbers and proportions of mothers who stopped breastfeeding within 17 weeks according to their breastfeeding score, as seen in Table 3.

## 3. Results

In the derivation and validation cohorts, we had 391 (83%), 633 (88%), and 579 (95%) mothers, respectively, with complete information on the included risk factors in the breastfeeding score. Among mothers in these cohorts, 194 (41%), 246 (39%), and 281 (49%) had stopped exclusive breastfeeding within 17 weeks after birth. A comparison of the distribution of the risk factors in the three cohorts showed that the educational level changed over time with an increasing proportion of mothers with a long-term education. Moreover, the age of the mothers at birth increased and fewer were smoking. Earlier breastfeeding duration among multipara only showed minor differences, whereas mothers in the validation cohort 2017 had reported similar levels of self-efficacy and sense of security towards breastfeeding as mothers in the derivation cohort 1999; mothers in the validation cohort 2004 reported slightly higher levels, as seen in Table 1.

Table 2 presents results of the Cox regression analysis in the three cohorts with log hazard ratios quantifying risk of breastfeeding cessation for each category relative to the reference category of the four risk factors. Scores assigned to each category of the four risk factors from the derivation cohort are also shown.

The ROC curves, seen in Figure 1, illustrate the association between sensitivity and specificity for different cut-points of the breastfeeding score, when mothers in the validation cohorts had been allocated points depending on their replies concerning the risk factors early after birth. The areas under the ROC curves were 0.77 (CI: 0.73–0.81) and 0.78 (CI: 0.74–0.82), respectively, for the validation cohorts from 2004 and 2017. A detailed calculation for the sensitivity and specificity with associated cut-points showed that a cut-point ≥5 gave the highest sensitivity and specificity. A cut-point ≥5 points predicted that 61% of first-time mothers and 42% of multiparous mothers in the validation cohort 2017 had a high risk of early breastfeeding cessation before 17 weeks with a sensitivity and specificity of 80% and 60% for first-time and 69% and 82% for multiparous mothers, respectively. The corresponding numbers for a cut-point ≥5 in the validation cohort 2004 were almost identical, pointing out that 60% of first-time and 41% of multiparous mothers were at risk; sensitivity and specificity were 74% and 53% for first-time mothers and 69% and 72% for multiparous mothers, respectively.

In both validation cohorts, the calibration plot with confidence intervals, as seen in Figure 2, showed a good agreement between the observed and predicted probability of breastfeeding cessation before the end of week 17 postpartum. From Table 3, it appears that 80% of first-time mothers and 69% of multiparous mothers with a risk score of 5–19 points stopped exclusive breastfeeding before 17 weeks in the validation cohort 2017. The corresponding numbers for mothers with a risk score of 5–19 points in the validation cohort 2004 were 68% and 70%, respectively.

## 4. Discussion

Validation of the revised version of the breastfeeding score in a general population of Danish mothers confirmed its ability to identify new mothers at risk of early breastfeeding cessation. The breastfeeding score combines four risk factors to a simple score. The score showed a high ability to discriminate between mothers with different risks and had a good predictive performance.

The updating of the breastfeeding score with a replacement of the sociodemographic risk factor from completed primary or secondary school to completed level of further education included recalibration of the relative weight of the four risk factors included in the breastfeeding score, as suggested by Moons et al. [18]. The use of retrospective data when testing the performance of the breastfeeding score made it possible not only to compare observed events of early breastfeeding cessation with the prediction from the breastfeeding score, but also to assess the ability of the breastfeeding score to discriminate and calibrate between risk groups and thereby assess the clinical usefulness of the score [18]. Self-reported data on the risk factors used to validate the breastfeeding score were collected early after birth independently of collection of data on the outcome of exclusive breastfeeding. Although mothers generally recall their breastfeeding duration quite accurately [24], the collection of data on breastfeeding duration by health professionals must be considered more reliable, which was the case in the derivation cohort. The transferability of the breastfeeding score was supported by the validation of the cohort from 2017, consisting of an unselected study population of new mothers living in different geographical areas of Denmark, with a diverse population of mothers reflecting the Danish background population according to age and level of education [25].

The four included risk factors in the breastfeeding score represented different domains of the multifactorial structure of breastfeeding. Level of completed education contributed with information on maternal sociodemographic background, well-known to influence breastfeeding duration [6]. Previous breastfeeding experience contributed with information on the pre and perinatal period, including first time motherhood and the success of breastfeeding for the multiparous mother expressed by the duration of exclusive breastfeeding of the previous child. Mothers tend to breastfeed for just as long as or a little longer than they did with a previous child [26]. Self-efficacy and sense of security concerning breastfeeding contributed with information on the psychosocial aspects of breastfeeding, which have been shown to be essential predictors of breastfeeding duration [7,19,27]. The general self-efficacy towards breastfeeding expressed the mothers’ confidence in overcoming any difficulties and achieving success [28]. Thereby, the predictor expressed maternal ability and strengths to overcome early problems and continue breastfeeding. Sense of security towards breastfeeding gathered information about the insecurity many mothers experience when they start breastfeeding. Concerns may be related to having enough milk [29] and getting used to a responsive feeding style with an infant-led approach [30,31]. Breastfeeding implies to learn how to read the infant’s cues on hunger and satiety to have a sense of security because the amount of milk the infant ingests is not visible. The inclusion of predictors from different domains of the multifactorial construct of breastfeeding may explain why the breastfeeding score maintains its predictive ability over time in different populations of new mothers.

The breastfeeding score indicated a high predictive value. The Self-efficacy Scale-Short Form (BSES-SF) has been found to predict the exclusive breastfeeding to 16 weeks in smaller study samples [12,14]. A direct comparison of the predictive values of the two screening tools are not possible, as we have not been able to find a comprehensive validation of the BSES-SF. The cut-point of five revealed a good ability to discriminate between subgroups by pointing to 60% of first-time mothers and 40% of multiparous mothers to be at risk of early breastfeeding cessation. The outcome of exclusive breastfeeding was determined based on mothers who stopped breastfeeding within 17 weeks, because this is the minimum recommended duration of exclusive breastfeeding according to the European Committees on Nutrition ESPGHAN and EFSA [32,33]. The relatively high proportion of mothers at risk of early breastfeeding cessation reflected the high proportion of mothers who generally stop exclusive breastfeeding before four months [1]. Cochrane reviews have repeatedly shown positive effects of supporting mothers in the breastfeeding act [5]. The breastfeeding score may ensure that the intervention is addressed to the insecure mothers who wish to breastfeed by pointing to mothers at risk of early cessation and in need for early support. From a public health perspective, it is especially important to identify the insecure first-time mothers and support them in establishing breastfeeding, considering the association between breastfeeding duration of the first and later infants [27].

From a practical point of view, the breastfeeding score is easy to use with a simple scoring system, which does not require technical assistance. The breastfeeding score aims to identify women with increased need for additional support when establishing breastfeeding. A short questionnaire can be used to obtain the breastfeeding score as it is based on self-reported questions. An example of a questionnaire is enclosed in Table 4. Because the breastfeeding score is self-reported, any health professional with an interest in identifying mothers in need of additional support when establishing breastfeeding can use the score. The time after discharge to home is the optimal time to screen the mother, since at that time she has experiences with breastfeeding and is therefore able to answer the questions more reliably in relation to her experiences.

The breastfeeding score is an addition to the health professional’s own assessment. The health professional often knows the educational level of the mother in advance, but less about the mothers’ experiences with and expectations to breastfeeding. When used in a Danish setting, health visitors describe the questions included in the screening tool as suitable to initiate and open a dialogue about how mothers experience breastfeeding and create basis for further guidance on areas of importance. Moreover, the simple scoring system connected to the answers of the questions makes it easy to calculate the mother’s score. A practical application with a key for reading the score is enclosed in Table 5. A cut-point of five will identify the first-time mother who expresses a low level of self-efficacy or sense of security towards breastfeeding, and, likewise, the multiparous mother with previous short breastfeeding experience. To prove these benefits, the breastfeeding score needs further process evaluation among health professionals using the breastfeeding score in their daily practice in different settings.

The validation of the breastfeeding score was carried out in Denmark, a country with a well-educated population and a long tradition for initiating breastfeeding after birth and for breastfeeding for a relatively long period of time [34]. There is no reason to believe the questions included in the breastfeeding score will be understood or perceived differently in other western societies with a culture similar to the Danish; however, additional studies are needed to confirm the prognostic value of using the breastfeeding score in other settings.

## 5. Conclusions

The breastfeeding score, consisting of four risk factors representing the sociodemographic, perinatal, and psychosocial domains of breastfeeding, showed a high predictive value and a good ability to discriminate between mothers at risk of exclusive breastfeeding cessation before 17 weeks postpartum. Both the temporal and external validation of the breastfeeding score supported the predictive value of the four questions asked shortly after birth. The construction with a simple score connected to the answers of the four questions makes the breastfeeding score easy to use in daily practice.

## Figures and Tables

**Table 1:** Characteristics of mothers in the derivation and validation cohorts with complete information on the four questions included in the breastfeeding score.

Characteristics	Derivation Cohort 1999	Validation Cohort 2004	*p* Value *	Validation Cohort 2017	*p* Value *
	*n* = 391	*n* = 633		*n* = 579	
	n (%)	n (%)		n (%)	
**Sociodemographic Factors**					
Maternal age: How old are you?					
17–23	36 (9)	43 (7)	0.32	34 (6)	0.002
24–31	245 (63)	391 (62)		323 (56)	
>32	109 (28)	196 (31)		222 (38)	
Vocational Education: Which vocational education have you completed?					
None–short-skilled	256 (65)	373 (59)	0.04	195 (34)	<0.001
Theoretical bachelor–master	135 (35)	260 (41)		384 (66)	
Smoking: Do you smoke now after giving birth?					
Yes	78 (20)	113 (18)	0.40	33 (6)	<0.001
No	313 (80)	520 (82)		546 (94)	
**Perinatal factors**					
Parity: How many children have you given birth to?					
Primipara	157 (40)	257 (41)	0.91	276 (48)	0.02
Multipara	233 (60)	376 (59)		303 (53)	
Previous breastfeeding experience: How many weeks did you breastfeed your previous child without giving it anything else?					
None, primipara	157 (40)	257 (41)	0.09	276 (48)	<0.001
0–5 weeks	31 (8)	40 (6)		70 (12)	
6–17 weeks	104 (27)	137 (22)		83 (14)	
>17 weeks	99 (25)	199 (31)		150 (26)	
**Psychosocial factors**					
Self-efficacy with respect to breastfeeding: How certain are you that you can complete four months of exclusive breastfeeding					
Medium to very uncertain (1–3)	131 (33)	153 (24)	<0.001	189 (33)	0.78
Certain to very certain (4–5)	260 (67)	480 (76)		390 (67)	
Sense of security not knowing the exact amount of milk the baby ingests: I feel fine about not knowing exactly how much milk my child gets when being breastfed					
Is not true (1–2)	181 (46)	240 (38)	0.01	246 (42)	0.24
Is fairly–exactly true (3–5)	210 (54)	393 (62)		333 (58)	

Note: *p* value * from a comparison with derivation cohort.

**Table 2:** Risk factors associated with breastfeeding cessation in the first 17 weeks after delivery.

Risk Factors	Prognostic Index						
	Derivation Cohort 1999			Validation Cohort 2004		Validation Cohort 2017	
	Log HR	95% CI	Scores	Log HR	95% CI	Log HR	95% CI
Completed Vocational Education							
None–short–skilled	0.32	−0.04–0.69	1	0.51	0.24–0.79	0.24	0.00–0.48
Theoretical bachelor–master	ref.	ref.	0	ref.	ref.	ref.	ref.
Previous breastfeeding experience:							
None, primipara	0.89	0.25–1.53	3	1.02	0.62–1.42	0.49	0.11–0.87
0–5 weeks	1.79	1.05–2.52	6	1.49	0.96–2.03	0.85	0.39–1.31
6–17 weeks	1.14	0.49–1.79	4	0.77	0.33–1.21	0.78	0.34–1.22
>17 weeks	ref.	ref.	0	ref.	ref.	ref.	ref.
Self-efficacy with respect to breastfeeding							
Uncertain	0.82	0.48–1.17	3	0.98	0.70–1.25	1.11	0.85–1.38
Certain	ref.	ref.	0	ref.	ref.	ref.	ref.
Sense of security not knowing the exact amount of milk the baby ingests							
Insecure	0.72	0.35–1.08	2	0.51	0.24–0.77	0.71	0.46–97
Secure	ref.	ref.	0	ref.	ref.	ref.	ref.

Cox regression analysis of data from 391 mothers with complete information in the derivation cohort, and assigned scores included in the breastfeeding score for each category of the four risk factors. For comparison, the corresponding Cox regression analyses of data from 633 and 579 mothers in the validation cohorts.

**Table 3:** Primiparous and multiparous mothers in the validation cohorts divided into risk groups according to their breastfeeding scores. Numbers and proportions of mothers who stopped breastfeeding within 17 weeks in each risk group.

Breastfeeding Score	Validation Cohort 2004			Validation Cohort 2017		
	n (%)	No. Stopped	% (95% CI)	n (%)	No. Stopped	% (95% CI)
Primipara						
0–4 points	103 (40)	32	31 (22–41)	109 (39)	29	27 (19–36)
5–19 points	154 (60)	92	60 (0.52–0.68)	167 (61)	113	68 (60–75)
Multipara						
0–4 points	222 (59)	38	17 (12–23)	177 (58)	43	24 (18–31)
5–19 points	154 (41)	84	55 (46–63)	126 (42)	96	76 (68–83)

**Table 4:** Practical application of the breastfeeding score. The written postpartum questionnaire for mothers.

The purpose of the questions is to give you the breastfeeding guidance that meet your needs *Just enter a number or cross in the box that best suits your situation*		
**Basic information**	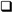	none / short / skilled
Which education have you completed?	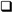	bachelor / master
**Your experiences with breastfeeding**		
This is my first child	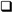	yes
If you have given birth earlier, how many weeks did you breastfeed your previous child without giving it anything else than your milk?	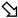	weeks
**Your expectations to breastfeeding**	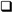	very uncertain
How certain are you that you can complete four months of exclusive breastfeeding?	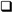	
	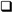	
	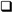	
	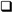	very certain
**How does the following apply to you?**	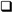	is not true
I feel fine about not knowing exactly how much milk my baby gets when being breastfed?	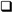	
	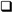	
	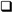	
	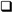	exactly true

**Table 5:** Practical application of the breastfeeding score. Key for reading the score.

When the mother has completed the questionnaire, her answers are converted into a total score. The individual questions are awarded points according to the system below.		
Completed vocational education	none / short / skilled	1
	bachelor / master	0
Previous breastfeeding experience	None, first-time mother	3
	0–5 weeks	6
	6–17 weeks	4
	>17 weeks	0
Self-efficacy with respect to breastfeeding	Medium to very uncertain, 1–3	3
	Certain to very certain, 4–5	0
Sense of security not knowing the exact amount of milk the baby ingests	Is not true, 1–2	2
	Is fairly–exactly true, 3–5	0
The points are added up to a total score		
Overall score: 0–5 = mothers are offered standard care		
Overall score: 6–19 = mothers are offered additional support during establishment of breastfeeding		
